# Hypothalamic Renin–Angiotensin System and Lipid Metabolism: Effects of Virgin Olive Oil versus Butter in the Diet

**DOI:** 10.3390/nu13020480

**Published:** 2021-01-31

**Authors:** Ana Belén Segarra, Germán Domínguez-Vías, José Redondo, Magdalena Martínez-Cañamero, Manuel Ramírez-Sánchez, Isabel Prieto

**Affiliations:** 1 Unit of Physiology, Department of Health Sciences, University of Jaén, Las Lagunillas, 23071 Jaén, Spain; asegarra@ujaen.es (A.B.S.); jrpunzano@gmail.com (J.R.); msanchez@ujaen.es (M.R.-S.); 2 Department of Physiology, Faculty of Health Sciences, Ceuta, University of Granada, 18071 Granada, Spain; germandv@go.ugr.es; 3 Unit of Microbiology, Department of Health Sciences, University of Jaén, Las Lagunillas, 23071 Jaén, Spain; canamero@ujaen.es

**Keywords:** virgin olive oil, brain renin–angiotensin system, fatty acid metabolism, energy balance

## Abstract

The brain renin–angiotensin system (RAS) has been recently involved in the homeostatic regulation of energy. Our goal was to analyse the influence of a diet rich in saturated fatty acids (butter) against one enriched in monounsaturated fatty acids (olive oil) on hypothalamic RAS, and their relationship with the metabolism of fatty acids. Increases in body weight and visceral fat, together with an increase in aminopeptidase A expression and reductions in AngII and AngIV were observed in the hypothalamus of animals fed with the butter diet. In this group, a marked reduction in the expression of genes related to lipid metabolism (LPL, CD36, and CPT-1) was observed in liver and muscle. No changes were found in terms of body weight, total visceral fat and the expression of hepatic genes related to fatty acid metabolism in the olive oil diet. The expressions of LPL and CD36 were reduced in the muscles, although the decrease was lower than in the butter diet. At the same time, the fasting levels of leptin were reduced, no changes were observed in the hypothalamic expression of aminopeptidase A and decreases were noted in the levels of AngII, AngIV and AngIII. These results support that the type of dietary fat is able to modify the hypothalamic profile of RAS and the body energy balance, related to changes in lipid metabolism.

## 1. Introduction

Obesity and overweight represent one of the most important health problems worldwide, being the sixth most important death risk factor. The increase in the amount of white adipose tissue in the body is determined by an imbalance between energy intake and energy expenditure. However, strategies for the prevention and management of obesity are usually based on controlling total energy intake and on increasing physical activity rather than on long-terms changes in the use of the energy by the body [1].

The high energy density characterizing high-fat diets (HFDs) is the main cause of the relationship between the consumption of this diet and overweight. HFDs have been used to develop obesity in laboratory animal models (Diet Induced Obesity (DIO) model) [2]. However, diet with a high percent of calories from fat, such as the Mediterranean diet, having around 35% of total energy from fat, have demonstrated a protective role against the development of cardiovascular diseases and obesity. This effect seems to be related to the high amount of monounsaturated fatty acids in its composition [3,4].

The renin–angiotensin system (RAS) is a complex peptide and enzyme system that has historically been implicated in the control of blood pressure and fluid homeostasis, but during the last decades, new insights into this system have been reported [5]. The local brain RAS has been demonstrated to participate in different physiological functions, including food intake and the body’s energy balance, in addition to its classic role in the control of water balance and maintenance of normal blood pressure [6,7,8,9].

The activity of the renin–angiotensin cascade in plasma or different tissues, including the brain, depends on the enzyme renin, which acts on the angiotensinogen to produce the peptide Ang I. The following steps in this cascade are determined by other peptidase activities. On the main system pathway, Ang I is metabolized to Ang II by the Angiotensin Converted Enzyme (ACE), and Ang II is metabolized to Ang III by the aminopeptidase A (EC 3.4.11.7). Finally, Ang III is transformed to Ang IV by the alanyl aminopeptidase (EC 3.4.11.2). Other alternatives pathways have been also proposed for the metabolism of angiotensin peptides. For example, Ang I and Ang II can be also metabolized to Ang 1–9 and Ang 1–7 by the angiotensin converting enzyme 2 (ACE2) and Ang I can be transformed to Ang 1–10 by aspartyl aminopeptidase (EC 3.4.11.21, AspAP) [10]. 

Drugs that decrease the synthesis of angiotensin II, or block its receptors have been proposed as a pharmacological treatment for hypertension for decades and, more recently, to prevent insulin resistance and obesity [11]. A relationship has been established between the peripheral and brain RAS and the energy storage in white adipocytes [9]. Cells of the arcuate nucleus (ARC) of the hypothalamus co-express the AngII receptor (AT1a) and the leptin receptor (Lepr). These cells are also required for the specific stimulation of thermogenic sympathetic nervous activity and the subset of changes in the resting metabolic rate but not for the inhibition of food intake, giving the hypothalamic RAS a relevant role in the control of the body’s energy balance [12]. Multiple physiological and environmental conditions, including diet, determine the proper functioning of this system. Although the role of brain RAS on the control of energy balance is well established, the possible relationship between brain RAS and changes in the metabolic use of fatty acid has not yet been studied.

The goal of the present study was to analyze if the effect of two high-fat diets, formulated with different lipid sources, on weight gain and adiposity in an animal model (male Swiss Webster ICR CD-1 mice) could mediate changes in hypothalamic RAS. We evaluated the alteration of fatty acid metabolism in the liver and skeletal muscle, with the objective to investigate if the disparity in fatty acid catabolism was associated with different fat depositions and leptin release that could explain the effect on hypothalamic RAS.

## 2. Materials and Methods

### 2.1. Animals and Experimental Design

Six-week-old male Swiss Webster ICR CD-1 mice (two series of *n* = 30, Harlan Laboratories, Barcelona, Spain) were divided into three experimental groups. The control group received a laboratory cereal-based chow diet (Panlab, Barcelona, Spain) with 3% fat, whereas the others two groups were fed two different high-fat diets. The Extra Virgin Olive Oil (EVOO) animals were provided the control diet supplemented with organic extra virgin olive oil (Soler Romero, Alcaudete, Spain) (20%), and the butter-fed mice were provided the control diet supplemented with commercial butter (Hacendado, Valencia, Spain) (20%). The HFDs were isoenergetic (35% kJ from fat) but hyperenergetic compared to the control diet (3% kJ from fat) and different in their fatty acids profile (EVOO: 78.6% monounsaturated fatty acids (MUFAs), 4.2% polyunsaturated fatty acids (PUFAs), and 17.1% saturated fatty acids (SFAs); butter: 35.6% MUFAs, 1.5% PUFAs, and 62.5% SFAs), and cholesterol and polyphenols amounts. Each group of animals received the corresponding diets ad libitum during an experimental period of 12 weeks. The animals had free access to tap water and were housed under controlled temperature (25 °C) and relative humidity (50% ± 5%), with a light/dark cycle of 12/12 h.

At the end of the experimental period, the animals were transferred to individual metabolic cages for 24 h, and the individual food intakes and body weights were measured to determine the metabolic balance. The day after, the mice were anesthetized with equithensin (42.5 g/L chloralhydrate dissolved in 19.76 mL ethanol, 9.72 g/L Nembutal, 0.396 L/L propylene glycol, and 21.3 g/L magnesium sulphate in distilled water) at a dose of 2 mL/kg body weight injected intraperitoneally. Blood samples was obtained from the left cardiac ventricle and transferred to centrifuge tubes with heparin as the anticoagulant, and the animals were perfused with saline solution. Total brain, liver, heart, gastrocnemius muscle, and visceral fat were dissected. Samples of the liver and gastrocnemius muscle were isolated and cooled in dry ice. The hypothalamus was dissected according to the stereotaxic Paxinos and Franklin atlas [13]. The blood samples were centrifuged at 5000× *g* for 5 min to obtain the plasma. All the samples (plasma, hypothalamus, liver, and muscle) were stored at −80 °C until use.

All experimental procedures were performed in accordance with the European Communities Council Directive 86/609/EEC and reviewed and approved by the Bioethics Committee of the University of Jaén, initially for project 2010/00047/001 (P.I. UJA_2009_Acción 13) and extended for project 2014/00215/001 (Acción 2_Puente_FT).

Plasmatic concentrations of leptin and ghrelin were measured according to the manufacturers’ protocols in a multianalyte profiling using the Luminex-100 system and the XY Platform (Thermo Fisher Scientific, Waltham, MA, USA) [14].

### 2.2. Determination of Aminopeptidase A Activity

Samples of the hypothalamus were used for the measurement of aminopeptidase A activity, estimated as glutamyl aminopeptidase activity. Each sample was homogenized in 500 µL Tris-ClH 50 mM buffer (pH 7.4) plus Triton-X-100 (1%), and centrifuged at 12,000× *g* at 4 °C for 10 min. To remove the detergent from the medium before determining the enzyme activity, the adsorbent polymeric Bio-beads SM-2 (Sigma-Aldrich, St. Louis, MO, USA, 100 mg/mL) was used, shaking the samples for 2 h at 4 °C. Supernatants were used for determination of the total amount of protein and glutamyl aminopeptidase activity [15]. Briefly, 20 µL of each supernatant was incubated with 100 µL substrate solution (2.72 mg L-glutamyl-β-naphthylamide, 10 mg dithiothreitol (DTT), and 10 mg bovine serum albumin (BSA) in 100 mL of 50 mM phosphate buffer, pH 7.4). The enzymatic kinetics were measurement over 60 min at 37 °C. The results of the fluorogenic assays were linear with respect to time of hydrolysis and protein content, and the activities are expressed as pmol of L-glutamyl-β-naphthylamide hydrolyzed per minute and per milligram of protein.

### 2.3. RNA Isolation and Real-Time PCR of Aminopeptidase A

Deep-frozen samples of mice hypothalamus were directly homogenized in TriPure isolation reagent (Bioline Iberia, Almería, Spain) using a hand homogenizer. Total RNA was extracted using a Direct-zol™ RNA MiniPrep Kit (Zymo Research, Irvine, CA, USA) according to the manufacturer’s protocol. Later, treatment with DNase (RNase-free) was performed following the manufacturer’s recommendations. The purity and concentration of total RNA were determined by measuring optical densities at 260/280 and 260/230 nm. First-strand complementary DNA was synthesized from 2 μg total RNA using a Maxima First Strand cDNA Synthesis Kit for RT-qPCR with dsDNase (Thermo Fisher Scientific, San Jose, MA, USA); negative control reactions were performed under the same conditions without reverse transcriptase.

Real-time PCR was performed with primers specific for mouse: β-actin: forward 5′-AGAGGGAAATCGTGCGTGAC-3′-, reverse 5′-CAATAGTGATGACCTGGCCGT-3′; APA: forward 5′-AGCGGAGATGCTTTTCTCAA-3′-, reverse 5′-ATGGAATGCAGAACTCTGGC-3′.

NT (no cDNA) and non-RT RNA (no RT enzyme) template reactions were used as the negative controls. All PCRs were performed at least three times, with three independent samples with the CFX384 Real-Time qPCR System (Bio-Rad, Madrid, Spain). The reactions were carried out as follows: 1 cycle of 1 min at 95 °C, 40 cycles at 95 °C for 10 s, 55 °C for 10 s, and 1 cycle of dissociation at 95 °C for 5 s. The melting point curves were checked to identify the final product. Relative quantifications were calculated with the standard curve method and normalization to the expression of the housekeeping gene coding the β-actin protein using the 2^−ΔΔCT^ method. Other housekeeping genes were discarded (Rn18s, GAPDH) because they showed significant variations with the high-fat diets.

### 2.4. Angiotensins UHPLC MS/MS

Deep-frozen hypothalamus aliquots (5 mg) were transfer to a 2-mL microcentrifuge tube, and the enzymatic activity of tissue proteases was inhibited by 20 s of microwave irradiation. To extract the peptides, the samples were homogenized in 500 µL acidified methanol solution (90% methanol and 9% glacial acetic acid) with a glass Potter-Elvehjem homogenizer. We added 5 µL of internal standard (Ang1–5) and 25 µL of Angiotensins Mix 20 ppm (100 µL of AngII 100 ppm, 100 µL of AngIII 100 ppm, 100 µL of AngIV 100 ppm, and 100 µL of AngI-7 100 ppm). All angiotensin peptides were purchased from Sigma-Aldrich (St. Louis, MO, USA). The samples were centrifuged at 1400× *g* for 25 min at 4 °C, and the supernatant was recovered in a centrifuge tube. The precipitate was resuspended in 300 µL of 1% trifluoroacetic acid and centrifuged at 1400× *g* for 25 min at 4 °C. The second supernatant was recovered in the same centrifuge tube as the first one. We transferred 500 µL of supernatant plus 500 µL 1% trifluoroacetic acid to a 1.5 mL centrifuge tube and centrifuged at the maximum speed for 20 min at 4 °C.

Solid-phase extraction (SPE) cartridges were washed three times with 500 µL of methanol and 1 mL of 1% trifluoroacetic acid water solution. The cartridges were rinsed with an 800 µL sample and then, after 2 min, were washed two times with 1 mL of 1% trifluoroacetic acid water solution. The analytes were eluted with 1 mL of 60% acetonitrile and 1% trifluoroacetic acid water solution. Finally, the eluents were frozen with dry ice and lyophilized until use.

Prior to UHPLC-MS/MS analysis, the samples were resuspended in 500 µL of 10 mM ammonium acetate and filtered with a 0.2 µm syringe filter (Sigma-Aldrich, St. Louis, MO, USA).

Angiotensin peptides were measured using a triple quadruple UHPLC-mass spectrometer (TSQ Quantum Access MAX model, Thermo-Science, Waltham, MA, USA).

Chromatographic separation was accomplished using an Acquity UPLC BEH C18 reversed phase column (50 × 2.1 mm, 1.7 µm) (Waters, Milford, MA, USA). The phase B mobile consisted of acetonitrile plus 0.1% formic acid, and the mobile A phase consisted of water plus 0.1% formic acid, delivered with a flow rate of 0.3 mL/min. The mobile phase gradient of solvent B was increased linearly to 16% B over the first 4 min, to 38% B at 8 min, to 50% at 10 min, and finally to 100% at 15 min. The column was re-equilibrated with solvent A before starting a new sample.

MS/MS conditions were as follows: capillary voltage of 6 kV, cone voltage of 20–25 V, de-solvation temperature of 350 °C, de-solvation gas flow of 600 L/h, and source temperature of 120 °C. The collision energies were set to 20 eV. Full-scan MS data were acquired in profile mode using Xcalibur software (Thermo-Science, Waltham, MA, USA).

### 2.5. RNA Isolation and Real-Time PCR of Genes Involved in Lipid Metabolism

Liver and muscle samples were used for the determination of expression by RT-qPCR of *LPL* (LipoProtein Lipase), CD36 (Cluster of Differentiation 36), CPT-1 (Carnitine Palmitoyl Transferase 1), and FAS (Fatty Acid Synthase) genes.

Deep-frozen samples of liver and muscle were extracted and purified using a Direct-zolTM RNA MiniPrep kit (Zymo Research, Irvine, CA, USA). A total of 50 mg of each sample was homogenized into 500 µL of TriReagent with a hand-held laboratory homogenizer (10–15 s) and centrifugated at 12,000× *g* for 10 min at 4 °C. The supernatants were mixed with 500 µ ethanol (95–100%) and centrifuged into columns at 13,000× *g* for 1 min at 4 °C. We added 400 µL RNA wash buffer to the columns, which were centrifuged another 30 s. We added 5 µL DNAsa and 75 µL digestion buffer to the columns, and they were incubated at room temperature for 15 min. We added 400 µL RNA PreWash to the columns, which were centrifuged twice at 13,000× *g* for 30 s at 4 °C. We added 700 µL RNA wash buffer to the columns, which were centrifuged at 13,000× *g* for 2 min at 4 °C. Finally, 100 µL of ultrapure water was added to the columns and centrifuged at 20,000× *g* for 1 min at 4 °C.

The purity of the total RNA was quantified by measuring optical densities at 260/280 and 260/230 nm.

First-strand complementary DNA was synthesized from 2 μg of total RNA using SensiFASTTM cDNA Synthesis (Bioline Iberia, Almería, Spain); negative control reactions were performed in the same conditions without reverse transcriptase.

Real-time PCR was performed with primers specific for mouse:

β-actin: forward 5′-AGAGGGAAATCGTGCGTGAC-3′, reverse 5′-CAATAGTGATGACCTGGCCGT-3′; *CD36*: forward 5′-AGCAACTGGTGGATGGTTTC-3′, reverse 5′-TCAAGGGAGAGCACTGGTTT-3′; *CTP-1*: forward 5′-TCCATGCATACCAAAGTGGA-3′, reverse 5′-TGGTAGGAGAGCAGCACCTT-3′; *FAS*: forward 5′-CATGACCTCGTGATGAACGTGT-3′, reverse 5′-CGGGTGAGGACGTTTACAAAG-3′; *LPL*: forward 5′-CTGGGCTATGAGATCAACAAGGT-3′, reverse 5′-AGGGCATCTGAGAGCGAGTCT-3′.

NT (no cDNA) and non-RT RNA (no RT enzyme) template reactions were used as the negative controls. All PCR setups were performed at least three times, with three independent samples. The reactions were carried out as follows: 1 cycle of 1 min at 95 °C, 40 cycle at 95 °C for 10 s, 55 °C for 10 s, and 1 cycle of dissociation at 95 °C for 5 s. Melting point curves were checked to identify the final product. Relative quantifications were calculated with the standard curve method and normalization to the expression of the housekeeping gene coding for the β-actin protein.

### 2.6. Statistical Analysis

Data analyses were conducted using the Statgraphics Centurion XVIII Software (Statgraphics Technologies, Inc., The Plains, VA, USA). All data were expressed as mean ± standard error of the mean (SEM). The statistical analysis for each variable were performed by one-way ANOVA analysis, followed by post hoc LSD test (Fisher’s least significant difference test) for pairwise comparisons between the three groups of animals. *p*-values below 0.05 were considered indicative of statistically significant differences.

## 3. Results

### 3.1. Effects of Diets on Body Weight and Total Visceral Adipose Tissue

Table 1 shows the mean values ± standard errors of the mean (SEMs) for daily food intake (g/day), body weight (g) at the end of the experimental period, and body mass index (BMI). Dissected heart, liver, and total visceral adipose tissue were expressed as grams and as percent of body weight in the three groups of animals.

No significant differences were found between the control and EVOO groups for daily food intake. However, at the end of the experiment, the nutter group showed significant lower food intake than the other two groups (3.2 ± 0.26, 3.4 ± 0.65, and 3.8 ± 0.41 g/day for the butter, control, and EVOO diets, respectively; *p* < 0.01). However, final body weight (44.12 ± 0.968, 40.54 ± 0.658, and 39.83 ± 0.261 g for the butter, control, and EVOO diets, respectively; *p* < 0.001), BMI (0.41 ± 0.010, 0.38 ± 0.005, and 0.38 ± 0.009; *p* = 0.1) and total visceral fat (0.95 ± 0.199, 0.40 ± 0.069, and 0.56 ± 0.074 for the butter, control, and EVOO diets, respectively; *p* < 0.05) were higher in the butter group than in the control and EVOO groups. The heart and liver weights did not show significant differences between the diets.

Table 2 shows the mean values ± SEMs for plasmatic levels of leptin and ghrelin in fasting and postprandial conditions (30 min after food intake). The data indicate significantly lower levels of leptin in the EVOO group during fasting (645.0 ± 80.11 vs. 1686.0 ± 166.33; *p* < 0.01) but not after food intake (3673.4 ± 476.03 vs. 1278.4 ± 149.85; *p* < 0.05). However, plasmatic leptin was highest in the butter group in the postprandial state (5485.1 ± 1932.35 vs. 1278.4 ± 149.85; *p* < 0.05). Significant differences were only achieved for plasmatic ghrelin between the EVOO and control and butter diets under the fasting condition (39.3 ± 9.29, 173.4 ± 56.06, and 31.6 ± 5.28 for the control, EVOO, and butter diets, respectively; *p* < 0.05). No differences were found after 30 min of food intake.

### 3.2. Hypothalamic Angiotensin Profile

**Figure 1:**
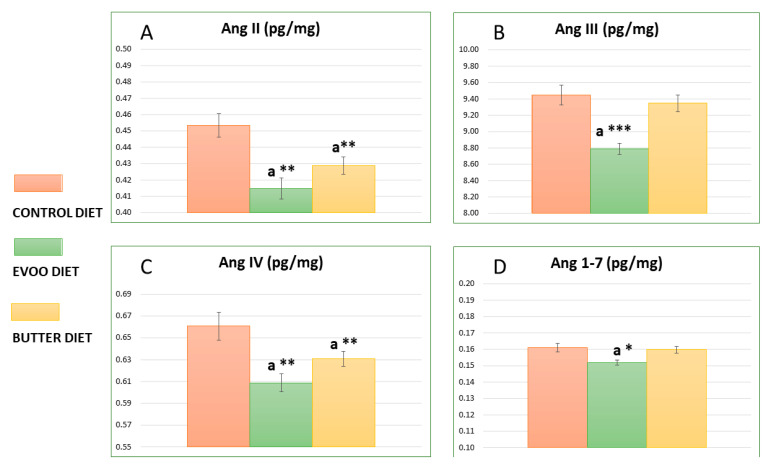
Means values ± SEMs obtained from the hypothalamus of experimental animals (the control, EVOO, and butter diets) for different angiotensin peptides (AngII (**A**), AngIII (**B**), AngIV (**C**), and Ang1–7 (**D**)) expressed as pg/mg. ^a^ Significant differences between the EVOO, butter, and control diets. * *p* < 0.05, ** *p* < 0.01, and *** *p* < 0.001.

Figure 1 represents the means values obtained from the hypothalamus of the experimental animals for different angiotensin peptides (AnII, AngIII, AngIV, and Ang1–7). The highest value in the control group was recorded for AngIII (9.45 ± 0.121), and the lowest was recorded for Ang1–7 (0.16 ± 0.003), similar to that for the EVOO and butter groups. The two high-fat diets had significantly lower amounts of AngII (0.41 ± 0.007 and 0.43 ± 0.005 vs. 0.45 ± 0.007 for EVOO, butter, and control, respectively; *p* < 0.01) and AngIV (0.60 ± 0.008 and 0.63 ± 0.007 vs. 0.66 ± 0.013 for EVOO, butter, and control, respectively; *p* < 0.01), but only the EVOO diet also showed lower AngIII (8.79 ± 0.067 vs. 9.45 ± 0.121; *p* < 0.001) and Ang1–7 levels (0.15 ± 0.152 vs. 0.16 ± 0.003; *p* < 0.05).

### 3.3. Hypothalamic Aminopeptidase A Activity and Expression

**Figure 2:**
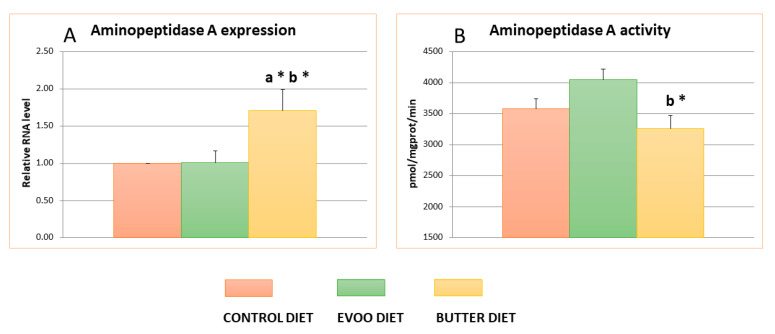
Means values ± SEMs of aminopeptidase A expression (**A**), as relative RNA level and aminopeptidase A activity (**B**), measured as glutamyl aminopeptidase activity, expressed as pmol of β-naphthylamine hydrolyzed per mg of protein and minutes in the hypothalamus of experimental animals (the control, EVOO, and butter diets). ^a^ Significant differences between EVOO, butter, and control. ^b^ Significant differences between EVOO and butter. * *p* < 0.05.

Figure 2 shows the mean values and standard errors for aminopeptidase A expression and activity (measurement as glutamyl aminopeptidase activity). No significant differences were found in the EVOO or butter groups compared to the control for aminopeptidase A activity. We observed a slight increase in the EVOO-diet animals and decrease in the butter-diet animals, which resulted in significantly lower activity levels in the butter group compared to the EVOO group. Nevertheless, no significant differences were found between the HFDs and the control group.

However, when aminopeptidase A expression was determined in the hypothalamic samples, we found that the expression levels were significantly higher in butter-fed animals, around 75% more, compared with the control and EVOO groups.

### 3.4. Hepatic Expression of Genes Involved in Lipid Metabolism

Table 3 shows the mean ± SEM of the mRNA expression of regulators of lipid metabolism in liver samples (relative RNA level). The expressions of LPL (1.01 ± 0.111), *CD36* (1.15 ± 0.116), and *CPT-1* (0.97 ± 0.133) were not affected by the inclusion of EVOO in the diet. However, the butter diet downregulated the expression of the *CPT-1* gene (0.68 ± 0.083; *p* < 0.05). Although they did not achieve significant differences, the mean values for *LPL* (0.73 ± 0.107) and *CD36* (0.72 ± 0.091) were lower compared with the control. The values of *CD36* gene expression were significantly lower in the butter than in the EVOO diets (0.72 ± 0.091 vs. 1.15 ± 0.116; *p* < 0.01). Finally, no differences were found in *FAS* gene expression between the three diets.

### 3.5. Skeletal Muscle Expression of Genes Involved in Lipid Metabolism

Table 4 shows the mean ± SEMs of the mRNA expression of key genes implicated in lipid metabolism in muscle samples. The butter diet downregulated the expression of the three genes involved in fatty acids catabolism (*LPL*, *CD36*, and *CPT-1*: 0.71 ± 0.090, 0.18 ± 0.038, and 0.35 ± 0.061, respectively; *p* < 0.01). The EVOO diet only significantly affected *LPL* and *CD36* gene expression, and the decreases were minor (0.71 ± 0.90 and 0.41 ± 0.088, respectively; *p* < 0.01). No significant changes were found for *FAS* gene expression for the butter or EVOO diets compared with the control, although mean values were higher in the EVOO group.

## 4. Discussion

High-fat diets (HFDs), which contain high amounts of saturated fatty acids and cholesterol, have been consistently related to undesirable changes in energy metabolism and the development of metabolic disorders, including hyperglycemia, type 2 diabetes, hypertriglyceridemia, nonalcoholic fatty liver disease, obesity, and hypertension [2,16]. The fat content of the diet seems to be an important factor in energy balance; usually, diets comprising more than 30% of total energy as fat produce obesity in animal models. However, the fatty acid profile seems to also be important in the control of total body weight and in the regulation of the number and volume of white adipocytes [17]. As such, unsaturated fatty acids were demonstrated to be less obesogenic than saturated fatty acids [18].

Our results indicated that only the animals fed the diet enriched with saturated fat (butter) showed significantly higher values of body weight and adiposity (total visceral fat) compared to control and EVOO diets, although the EVOO diet contained the same energy density, and that no marked differences were recorded in food intake although a significant reduction in food intake was established in the butter diet at the end of the experimental period, probably as a response to the increase in body weight. This beneficial effect of olive oil in the regulation of body weight has been described in previous works [19,20,21,22] and could be related to the minor components included in the unsaponifiable fraction of EVOO [23] and gut dysbiosis [24,25].

HFDs also affect the plasmatic levels of peptides involved in the control of food intake (leptin and ghrelin), both in the fasting and postprandial conditions. The consequences of our two hypercaloric diets were different. The EVOO diet diminished the levels of leptin but increased the levels of ghrelin during fasting. The butter diet only significantly affected plasmatic postprandial leptin, producing the highest values for this peptide. Chronic consumption of an HFD alters the plasma amounts of food-intake-regulating peptides. Cano et al. [26] reported the effect of a diet enriched with corn oil on the 24 h pattern of several adipokines in rats, showing a significant increase in leptin and a decrease in ghrelin compared with the chow diet. These changes in leptin and ghrelin have been related to a disruption of appetite regulatory receptors in vagal afferents [27]. Unexpectedly, our results showed that the EVOO diet decreased the amount of leptin and increased ghrelin during fasting. These results agree with previous data indicating that the supplementation of a HFD with an olive leaf extract avoided the increase in body weight and plasma leptin in mice [28], demonstrating once again the important protective role of the minor components of EVOO [29].

The increased plasma leptin with obesity has been related to changes in the brain RAS and the activity of the sympathetic nervous system, which determine the development of hypertension and metabolic alterations [30]. ASrAogen transgenic rats, with decreased glial expression of angiotensinogen, do not develop hypertension and obesity during aging, but its plasma levels of leptin are lower than in normal rats [31]. Lower levels of AngII in the brain have a protective effect on the development of obesity and the related metabolic impairments in rats fed HFDs; hence, RAS blockers were proposed as a pharmacological treatment for obesity [32]. Besides AngII, other peptides of the brain RAS have been implicated in the development and prevention of obesity, such as Ang1–7 [33].

The peripherical RAS is considered only part of this total system. The discovery of an entire RAS pathway in the brain has improved our understanding of how RAS influences the central nervous system [34,35,36,37]. Cells of the arcuate nucleus of the hypothalamus (ARC) colocalize the AngII receptor AT1a with the leptin receptor. This AT1 receptor is required for stimulation of thermogenic sympathetic nervous activity, regardless of changes in food intake or blood pressure [12]. This local hypothalamic system presents a divergent profile compared with the peripherical RAS system [10].

Our analysis of hypothalamic RAS showed that both HFDs significantly decreased the levels of AngII and AngIV; this effect was more marked for the EVOO diet. Only the diet supplemented with EVOO significantly diminished the amount of AngIII, whereas the butter diet maintained the same levels of AngIII as the control group, together demonstrating a significant increase in expression of the aminopeptidase A gene. However, no significant differences were found for aminopeptidase A activity when it was assayed as glutamyl aminopeptidase A activity. The differences found between the expression and the activity of aminopeptidase A may in part be due to posttranscriptional factors. In fact, we previously reported discrepancies between the expression of aminopeptidase A and its activity [38]. the possible influence of surrounding biochemical factors, such as changes in cholesterol and/or steroid levels, on this activity was hypothesized [39].

Although AngII was classically considered the main peptide in peripheral RAS, AngIII was demonstrated to also play a crucial role in the brain system; it was identified as the major effector peptide of the brain RAS in the control of blood pressure and vasopressin release [40,41,42]. AngII is quickly metabolized to AngIII by aminopeptidase A activity—one enzyme widely distributed in the plasma and several tissues, including the hypothalamus [20,43,44,45]. Previous works reported high levels of aminopeptidase A in the plasma of ob/ob obese mice [46] and prediabetic obese Zucker rats [47]. Conversely, a relation between this activity with the saturation of the dietary fatty acids has been established [48,49]. Our results support the hypothesis that lower levels of fasting leptin in plasma are related to the lower activity of several components of the hypothalamic RAS and halting of the development of obesity in the EVOO group. Conversely, animals fed the butter diet presented higher levels of postprandial leptin in serum and increased hypothalamic expression of aminopeptidase A, with similar levels of AngIII as in the control group, and an increased amount of total visceral fat compared with EVOO and control animals.

The hypothalamus plays a regulatory role of lipid metabolism in the liver through the sympathetic autonomic system; impairments of these pathways may contribute to dyslipidemia and hepatic steatosis [50,51]. The peripherical infusion of AngII in rats reduced the body weight and the amount of adipose tissue, whereas the intracerebroventricular infusion (icv) of RAS and β-adrenergic blockers prevented lipolysis and weight loss [52].

Liver and skeletal muscle, together white fat tissue, are the main sites for the control of the adaptive metabolic regulation of fatty acids in the body, playing a key role in the control of lipid homeostasis. Our results indicated that the chronical intake of saturated HFD (the butter group) decreased the expressions of *LPL*, *CD36*, and *CPT-1* in the liver and muscle, whereas monounsaturated HFD (the EVOO group) only decreased *LPL* and *CD36* gene expression in the muscle. Lipoprotein lipase activity plays an important role in lipid metabolism by hydrolyzing core triglycerides from circulating chylomicrons and very low-density lipoproteins (VLDLs). It is diminished by HFDs and restored after the inclusion of antioxidants in the diet [53,54].

The scavenger receptor *CD36* is a surface glycoprotein known as fatty acid translocase (FAT). *CD36* is a key player in fatty acid metabolism; it has been widely studied for its role in long-chain fatty acid uptake and oxidation [55]. *CD36* has been implicated in dysregulated fatty acid and lipid metabolism in pathophysiological conditions, particularly in HFDs. Squalene, a minor component of virgin olive oil, was reported to reduce the expression of *CD36* in monocytic cells and macrophages in vitro [56]. Finally, carnitine palmitoyl transferase 1 (*CPT-1*) is a rate-limiting enzyme that facilitates the transport of long-chain fatty acids into the mitochondria for β-oxidation, and it enhances insulin action in the muscle of high-fat diet and insulin-resistant rats [57].

Our results indicate that the EVOO diet, despite being an HFD, has a minor impact over fatty acid metabolism in liver and skeletal muscle, resulting in lower fat deposition and leptin release. The effect of the EVOO diet on brain RAS, with a lower expression of aminopeptidase A and levels of angiotensin peptides, could explain in part the beneficial effect of this diet on the maintenance of long-term body weight.

## 5. Conclusions

Taken together, our results suggest a differential effect of two high-fat diets on the development of obesity. The butter diet resulted in significant increases in body weight and total visceral adipose tissue, with high levels of postprandial leptin, in the stimulation of hypothalamic RAS and a decreased expression of proteins implicated in fatty acids catabolism in liver and muscle. The inclusion of extra virgin olive oil in diets decreased the levels of fasting leptin and moderated the activity of hypothalamic RAS, together with a minor effect on muscle and liver fatty acid metabolism and a suitable maintenance of body weight.

## Figures and Tables

**Table 1:** Mean values ± standard errors of the mean (SEMs) of daily food intake (g/day), finally body weight (BW, g), and Body Mass Index (BMI) and heart (HW), liver (LW), and total visceral adipose tissue (VATW), expressed as grams and percentage of body weight (HW/BW, LW/BW, and VATW/BW) for the three groups of animals (the control diet, the butter diet, and the Extra Virgin Olive Oil (EVOO) diet). a: significant differences between the EVOO, butter, and control diets. b: significant differences between EVOO and Butter.3.2. Leptin and Ghrelin Fasting and Postprandial Plasma Levels.

	CONTROL DIET	EVOO DIET	BUTTER DIET	
Body Weight (BW.g)	40.5 ± 0.66	39.8 ± 0.26	44.1 ±0.97 ^ab^	*p* < 0.01
Body Mass Index (BMI)	0.38 ± 0.005	0.39 ± 0.009	0.41 ± 0.010	ns
Heart weight (HW, g)	0.22 ± 0.005	0.22± 0.006	0.24 ± 0.009	ns
HW/BW (%)	0.54 ± 0.007	0.53± 0.014	0.55 ± 0.01	ns
Liver Weight (LW.g)	1.54 ± 0.075	1.75 ± 0.072	1.67 ± 0.065	ns
LW/BW (%)	3.79 ± 0.145	4.32 ± 0.148	3.88 ± 0.089 ^ab^	*p* < 0.05
Visceral Adipose Tissue Weight (VATW.g)	0.40 ± 0.069	0.56 ± 0.074	0.95 ± 0.199 ^ab^	*p* < 0.05
VATW/BW (%)	1.08 ± 0.184	1.39 ± 0.183	2.13 ± 0.401 ^ab^	*p* < 0.05
Food Intake (g/day)	3.4 ± 0.65	3.8 ± 0.41 ^a^	3.2 ± 0.26 ^ab^	*p* < 0.05

**Table 2:** Mean values ± SEMs expressed as pg/mL of plasmatic levels of leptin and ghrelin in fasting and postprandial conditions (30 min after food intake) for the three groups of animals (control diet, butter diet, and EVOO diet). a: significant differences between EVOO, butter, and control diets. b: significant differences between EVOO and butter.

	CONTROL DIET	EVOO DIET	BUTTER DIET	
Fasting leptin (pg/mL)	1686.0 ± 166.33	645.0 ± 80.11 ^a^	1646.0 ± 231.20 ^b^	*p* < 0.01
Postprandial leptin (pg/mL)	1278.4 ± 149.85	3673.4 ± 476.03	5485.1 ± 1932.35 ^a^	*p* < 0.05
Fasting ghrelin (pg/mL)	39.3 ± 9.29	173.4 ± 56.06 ^a^	31.6 ± 5.28 ^b^	*p* < 0.05
Postprandial ghrelin (pg/mL)	10.4 ±2.68	8.8 ± 0.76	9.8 ± 2.89	ns

**Table 3:** Mean values ± SEMs of mRNA expression (relative RNA level) of different genes regulators of lipid metabolism (CD36: cluster of differentiation 36; FAS: fatty acid synthase; CPT-1: carnitine palmitoyltransferase I; and PLP: lipoprotein lipase) in liver samples of the three groups of animals (the control, butter, and EVOO diets). a, b: significant differences between EVOO, butter, and control and between EVOO and butter, respectively. * *p* < 0.05 and ** *p* < 0.01.

	CONTROL DIET	EVOO DIET	BUTTER DIET
CD36	1	1.15 ± 0.116	0.72 ± 0.091 ^b^**
FAS	1	0.99 ± 0.111	1.03 ± 0.071
CPT-1	1	0.97 ± 0.133	0.68 ± 0.083 ^a^*
LPL	1	1.01 ±0.111	0.73±0.107

**Table 4:** Mean values ± SEMs of mRNA expression (relative RNA level) of different genes regulators of the lipid metabolism in skeletal muscle samples of the three groups of animals (the control, butter, and EVOO diets). a: significant differences between EVOO, butter, and control. b: significant differences between EVOO and butter. ** *p* < 0.01.

	CONTROL DIET	EVOO DIET	BUTTER DIET
CD36	1	0.41 ± 0.088 ^a^**	0.18 ± 0.038 ^a^**^b^**
FAS	1	1.33 ± 0.121	0.83±0.135 ^b^**
CPT-1	1	1.00 ± 0.114	0.35± 0.061 ^a^**^b^**
LPL	1	0.71 ± 0.090 ^a^**	0.59 ± 0.112 ^a^**

## Data Availability

Not applicable.
